# Bortezomib-induced neurotoxicity in human neurons is the consequence of nicotinamide adenine dinucleotide depletion

**DOI:** 10.1242/dmm.049358

**Published:** 2022-12-08

**Authors:** Andrew R. Snavely, Keungjung Heo, Veselina Petrova, Tammy Szu-Yu Ho, Xuan Huang, Crystal Hermawan, Ruth Kagan, Tao Deng, Ilyas Singeç, Long Chen, Lee B. Barret, Clifford J. Woolf

**Affiliations:** ^1^ F.M. Kirby Neurobiology Center, Program in Neurobiology, Boston Children's Hospital, Boston, MA 02115, USA; ^2^ Department of Neurobiology, Harvard Medical School, Boston, MA 02115, USA; ^3^ National Center for Advancing Translational Sciences (NCATS), Division of Preclinical Innovation, Stem Cell Translation Laboratory (SCTL), National Institutes of Health (NIH), Rockville, MD 20850, USA

**Keywords:** Chemotherapy-induced peripheral neuropathy, Neuropathy, Wallerian degeneration

## Abstract

The proteosome inhibitor bortezomib has revolutionized the treatment of multiple hematologic malignancies, but in many cases, its efficacy is limited by a dose-dependent peripheral neuropathy. We show that human induced pluripotent stem cell (hiPSC)-derived motor neurons and sensory neurons provide a model system for the study of bortezomib-induced peripheral neuropathy, with promising implications for furthering the mechanistic understanding of and developing treatments for preventing axonal damage. Human neurons in tissue culture displayed distal-to-proximal neurite degeneration when exposed to bortezomib. This process coincided with disruptions in mitochondrial function and energy homeostasis, similar to those described in rodent models of bortezomib-induced neuropathy. Moreover, although the degenerative process was unaffected by inhibition of caspases, it was completely blocked by exogenous nicotinamide adenine dinucleotide (NAD^+^), a mediator of the SARM1-dependent axon degeneration pathway. We demonstrate that bortezomib-induced neurotoxicity in relevant human neurons proceeds through mitochondrial dysfunction and NAD^+^ depletion-mediated axon degeneration, raising the possibility that targeting these changes might provide effective therapeutics for the prevention of bortezomib-induced neuropathy and that modeling chemotherapy-induced neuropathy in human neurons has utility.

## Teaser

**Summary:** Human stem cell-derived neurons confirm a role for Wallerian-like degeneration in bortezomib-induced axonal neurotoxicity.

## INTRODUCTION

Chemotherapy remains the mainstay treatment of many solid tumors, despite recent advances in cancer immunotherapy. However, the use of most chemotherapeutic agents is complicated by a high incidence of peripheral neuropathy that is independent of their anticancer mechanisms of action. On average, patients who develop chemotherapy-induced peripheral neuropathy (CIPN) receive less chemotherapy, incur higher healthcare costs and have a lower quality of life (Bhatnagar et al., 2014; Mols et al., 2014; Pike et al., 2012). One chemotherapeutic agent whose utility is substantially limited by the peripheral neuropathy commonly associated with it is bortezomib (BTZ). BTZ is a proteasome inhibitor that is the first-line treatment for multiple myeloma and mantle cell lymphoma, and BTZ-induced peripheral neuropathy (BIPN) is estimated to occur in 37-44% of patients (Grisold et al., 2012). Most commonly, BIPN presents as a painful sensory neuropathy in the distal extremities; however, extreme cases include motor and autonomic involvement (Velasco et al., 2019).

Despite neuropathy being the most common dose-limiting non-hematologic side effect of BTZ, there remain no therapies to treat or prevent BIPN. This is due to an incomplete understanding of the mechanisms by which chemotherapeutic agents that are selected for activity against highly proliferative cells damage post-mitotic neurons. Although the proximal causes of nerve damage remain subject to active debate, it is well established that the symptoms are the result of a dying-back degeneration of peripheral nerves. Plantar skin biopsies from mouse models of BIPN and patients with BIPN show a decreased density of unmyelinated nerve fibers invading the epidermis – including intraepidermal nerve fibers and bulbous encapsulated Meissner's corpuscle nerve endings (Bobylev et al., 2017; Bruna et al., 2010; Richardson et al., 2009). Nerve conductance studies in patients with peripheral neuropathy reveal a decrease in conduction velocity and amplitude, consistent with an axonal neuropathy (Chaudhry et al., 2008; Richardson et al., 2009). Given the central role of axon degeneration in BIPN, one approach to identifying novel treatments for the disease might be to understand the molecular mechanisms driving the destruction of the axon, enabling development of neuroprotective treatments, rather than targeting the insults that initiate this process, which might include their desired activity on cancer cells.

Growing evidence in mouse models of BIPN suggest that the Wallerian degeneration pathway that occurs distal to an axotomy might also play an important role in BTZ-induced axon degeneration (Geisler et al., 2019a). In contrast to developmental axon pruning, Wallerian degeneration is a caspase-independent pathway causing degradation of mature axons following a traumatic insult (Finn et al., 2000; Simon et al., 2012). First described in the context of nerve transection, Wallerian degeneration is regulated by the antagonistic activities of an axonal nicotinamide adenine dinucleotide (NAD^+^)-synthesizing enzyme, NMNAT2, and a NAD^+^-degrading enzyme, SARM1 (Conforti et al., 2014). Activation of SARM1 initiates a stereotyped cascade of events beginning with depletion of NAD^+^. The axon then enters a state of energetic crisis, involving reduction in cellular ATP and depolarization of the mitochondrial membrane. This energy crisis causes an elevation of axonal calcium and the activation of cellular proteases such as calpains. The consequent degradation of cellular proteins leads to axonal fragmentation, proceeding in a distal-to-proximal manner (Yang et al., 2013). In mice, BTZ induces both degradation of NMNAT2 and subsequent depletion of NAD^+^ (Geisler et al., 2019a). Moreover, SARM1 knockout is protective against BTZ-induced neuropathy in mouse models of the disease (Geisler et al., 2019a). These findings raise the question of whether inhibition of Wallerian degeneration might be beneficial in patients at risk for the development of BTZ-induced neuropathy.

In this study, we examine the extent to which neurons derived from human induced pluripotent stem cells (hiPSCs) recapitulate the neuronal responses to BTZ found in mouse models, with the goal of developing a disease model for drug screening and mechanistic studies. We demonstrate that hiPSC-derived neurons recapitulate many features of BTZ-induced neurotoxicity previously described in rodents, including effects on mitochondrial respiration and depletion of cellular NAD^+^. Moreover, BTZ-induced toxicity in human neurons is NAD depletion dependent and caspase activation independent. However, unlike rodent models, inhibition of SARM1 did not have a protective effect in human neurons.

## RESULTS

### BTZ-induced axon degeneration in hiPSC-derived neurons

**Fig. 1.**
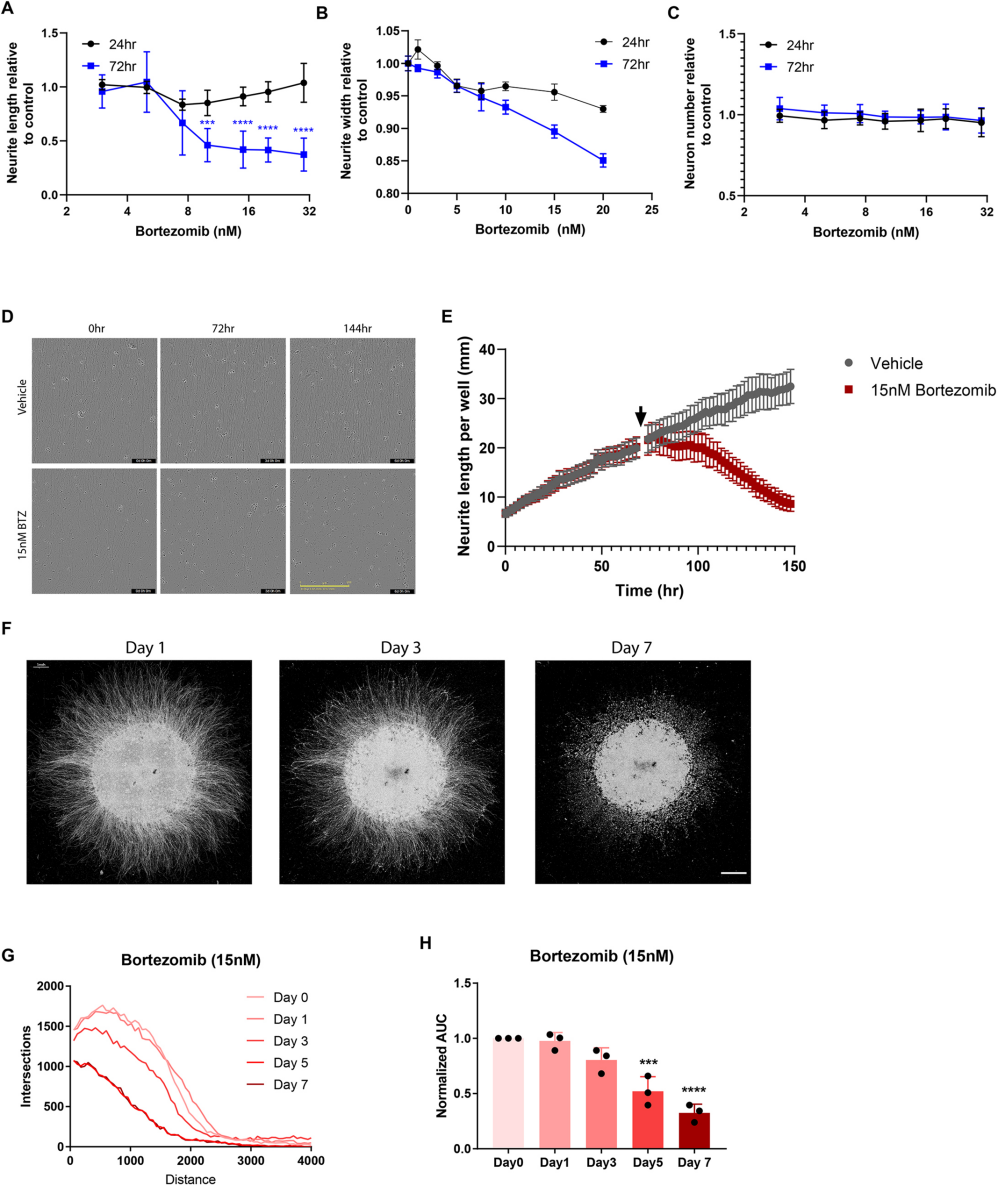
**Bortezomib-induced axon degeneration in hiPSC-derived neurons.** (A-C) hiMNs were treated with varying doses of bortezomib (BTZ) and fixed after 24 or 72 h. Neurons were stained for βIII-tubulin and imaged using high-throughput automated imaging at the indicated time points. Neurite length per neuron (A), neurite width (B) and neuron number (C) were calculated using automated neuron tracing software. Data were tested with a two-way ANOVA. Significant effects were observed for neurite length; treatment *F* (7, 236)=32.03, *P*<0.0001; time *F* (1, 236)=191.1, *P*<0.0001; and interaction *F* (7, 236)=21.90, *P*<0.0001; post hoc Sidak's multiple-comparisons test; DMSO versus BTZ 72 h (blue asterisks) (*n*=16). (D) Representative phase-contrast images showing the progressive morphological changes in hiMN neurites treated with vehicle or 15 nM BTZ. (E) hiMNs were imaged by phase-contrast microscopy every 4 h from the time of plating. Cells were treated with vehicle or 15 nM BTZ at 72 h (arrow). Neurite length was tracked over time using automated neurite tracing. (F) Representative images of hiMN spot cultures at 1, 3 and 7 days after treatment with 15 nM BTZ. (G) Plot showing the number of neurites present at varying distances (in µm) from the cell body cluster for the spot culture shown in C. (H) Quantification of axon degeneration in spot cultures by area under the curve (AUC) measurements. Values are shown as average (bars) and individual (dots) AUC values at 1, 3, 5 and 7 days after BTZ treatment. Data were compared with values at day 0 by one-way ANOVA with Dunnett's post hoc test. All error bars represent s.e.m. ***P<0.0001; ****P<0.00001. Scale bars: 200 μm (D); 1 mm (F).

In rodent neurons, BTZ induces axonal loss of delayed onset, starting between 24 and 36 h after treatment (Geisler et al., 2019a). To determine whether a similar delayed onset degeneration occurs in human neurons, hiPSCs were differentiated into ISL1-expressing motor neurons using a small-molecule-based differentiation method (Fig. S1A,B) (Wainger et al., 2014) and BRN3A^+^ sensory neurons (Deng et al., 2022 preprint) as described later. Purified cultures of hiPSC-derived motor neurons (hiMNs) were treated for 24 or 72 h with varying doses of BTZ, the cultures fixed, and neurite length measured using an automated neurite tracing software (see Materials and Methods). After 24 h, there was no significant change in neurite length between BTZ-treated and vehicle-treated hiMNs (Fig. 1A). However, after 72 h of BTZ exposure, neurons displayed a clear dose-dependent decrease in neurite length, starting at 7.5 nM BTZ and reaching a maximum effect at 10-15 nM. Although it is difficult to predict the concentration of BTZ at the affected peripheral nerves in patients, these concentrations are similar to those reported in the plasma of patients receiving BTZ, which range from 28 nM to 546 nM (Leveque et al., 2007). Consistent with reports that BTZ-induced neuropathy in patients and rodent models results from an axonopathy and not a neuronopathy, there was no change in the number of neurons across all the doses tested (Fig. 1B). These data indicate that BTZ causes a selective loss of neurites in hiMNs.

BIPN is considered a dying-back neuropathy, beginning at the distal end of axons, and then progressing towards the cell body. However, as the neurites of hiMNs are actively growing, it is impossible to differentiate a deficit in neurite outgrowth from neurite degeneration in static images of fixed cultures. To demonstrate whether BTZ treatment induces a loss of established neurites, we performed longitudinal live-cell imaging of hiMNs. After 3 days, BTZ-treated neurons showed a clear decrease in neurite diameter and increase in axonal swellings, consistent with the morphological changes that occur in axon degeneration (Fig. 1C). Using automated neurite tracing, we quantified neurite mass in hiMN cultures at frequent intervals (every 4 h) over a 6-day period, treating with either vehicle or 15 nM BTZ on day 3. Vehicle-treated neurons continued to grow at a relatively constant rate throughout the imaging period (Fig. 1D). In contrast, neurons treated with 15 nM BTZ showed an initial decrease in further neurite outgrowth, but minimal decrease in neurite length. This period of relative growth stasis lasted approximately 24 h, consistent with our finding that BTZ had little effect on neurite length in cultures fixed at 24 h. After 24 h, however, the BTZ-treated cultures entered a phase of rapid neurite loss, which continued at a steady rate for 48 h before beginning to level off. Thus, live imaging with a high acquisition rate uncovered characteristics of the hiMN response to BTZ that were not appreciable with fixed cultures, including both an initial period of growth arrest and then axon die back.

Having confirmed that BTZ induces axon loss in hiMNs, we next asked whether this degeneration occurred in a distal-to-proximal manner, as expected for BIPN. We developed a method for growing hiMNs in spot cultures, using methods similar to those used previously for primary dorsal root ganglion cells (Latremoliere et al., 2018). The radial orientation of neurites extending from the spots allows for the distal and proximal axon to be distinguished in a manner impossible in dissociated cultures. Addition of 15 nM BTZ caused a clear degeneration of all neurites over a period of 7 days (Fig. 1E). We performed a modified version of a Sholl analysis at days 0, 1, 3, 5 and 7 to determine the distribution of neurite lengths in the spot cultures over time (Fig. 1F-H). The progressive collapse over time in the number of neurites present at increasingly shorter distances from the cell body is indicative of a degeneration beginning at the more distal portions of the neurites and then progressing towards more proximal regions, exactly as expected in an axon dying-back process.

### Mitochondrial dysfunction coincides with neurite loss in BTZ-treated hiPSC-derived neurons

**Fig. 2.**
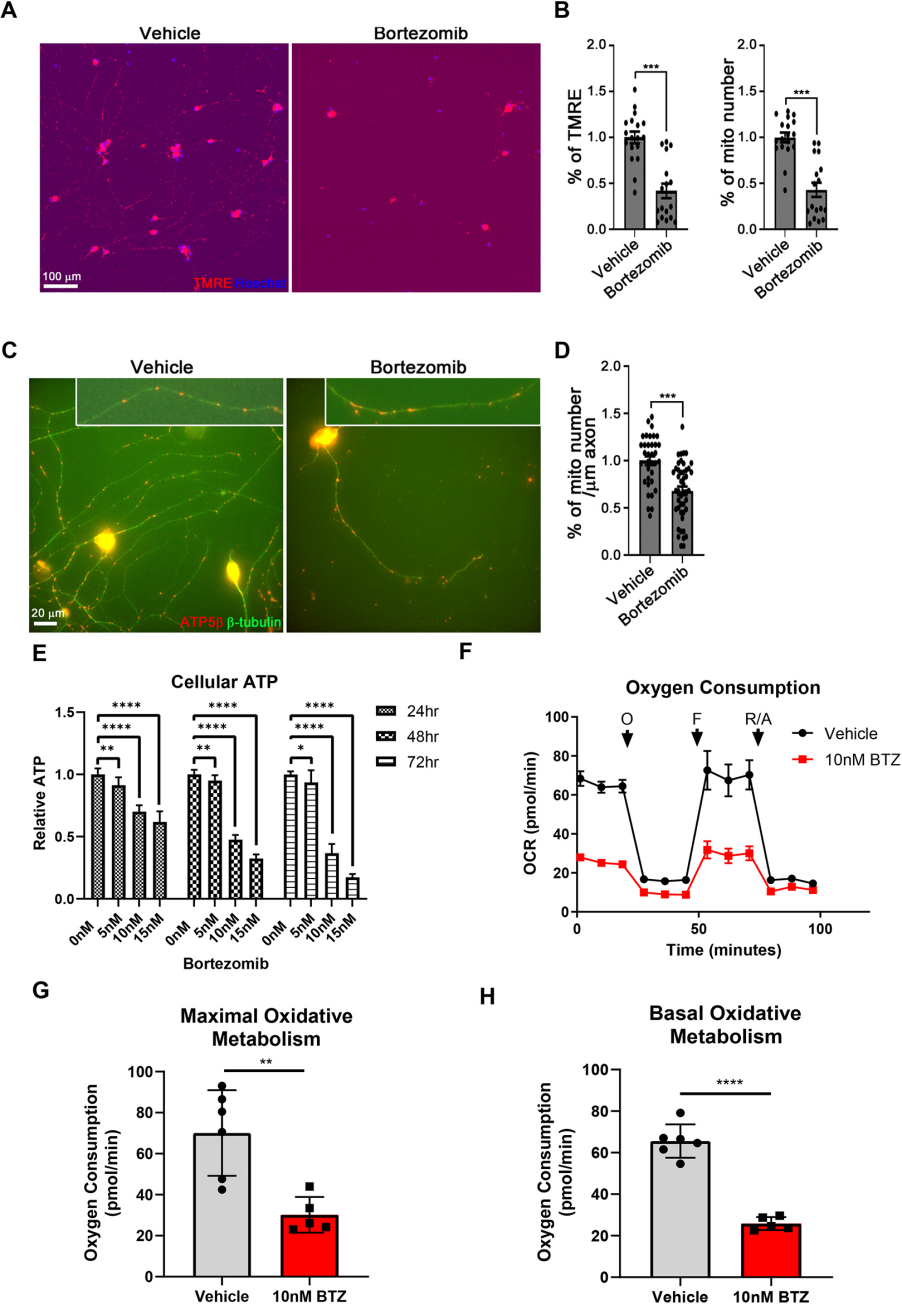
**Mitochondrial dysfunction is an early event in bortezomib-induced axon degeneration.** (A) Representative images of TMRE-labeled mitochondria within cell bodies, dendrites and axons (red) and Hoechst 33342 (blue) in vehicle and 10 nM BTZ treated hiMNs. Scale bar: 100 μm. (B) Quantification of the percentage of TMRE and mitochondria number in vehicle- and 10 nM BTZ-treated hiMNs. Statistical measurements were done with two-tailed unpaired Student's *t*-test. ***P<0.001. All error bars represent s.e.m. Data are from three independent cultures. (C) Representative images of mitochondria in vehicle- and 10 nM BTZ-treated neurons. The mitochondria within 100 μm distal axons are shown in boxes. Mitochondria was stained with ATP5β (red) and dendrites and axons were stained with βIII-tubulin (green). The experiments were performed in three independent cultures. Scale bar: 20 μm. (D) Quantification of the mitochondrial number per micrometer of axon. The mitochondria were measured from axon per neuron. Statistical measurements were done with two-tailed unpaired Student's *t*-test. ***P<0.001. All error bars represent s.e.m. (E) hiMNs were treated with varying doses of BTZ and subjected to luminescence-based assays for ATP at 24, 48 or 72 h. Data were tested with a one-way ANOVA with Dunnett's multiple-comparisons test. *P<0.05; **P<0.005; ***P<0.0005; ****P<0.00005; (*n*>14). (F) Oxygen consumption rates (OCR), as measured by Agilent Seahorse assay, in hiMNs treated with vehicle or 10 nM BTZ. Arrows represent the addition of oligomycin (‘O’), carbonyl cyanide-4 (trifluoromethoxy) phenylhydrazone (‘F’), and rotenone/antimycin (‘R/A’). (G,H) Quantification of average maximal (G) and basal (H) oxygen consumption. Data were tested with a two-tailed unpaired Student's *t*-test. **P<0.005; ****P<0.00005. (*n*>5).

Rodent models have demonstrated mitochondrial dysfunction, with both a loss of mitochondrial polarity and an energy crisis, as common features of multiple forms of chemotherapy-induced neuropathy, including BIPN (Canta et al., 2015; Cavaletti et al., 2007; Geisler et al., 2019a; Zheng et al., 2012). We employed tetramethylrhodamine, ethyl ester (TMRE) labeling to assess mitochondrial membrane potential following BTZ treatment. hiMNs were labeled with TMRE, a cell-permeant fluorescent dye that selectively labels mitochondria based on their membrane potential, and with Hoechst 33342 to identify cell bodies. The cells were imaged and the TMRE-labeled mitochondria in dendritic and axonal regions were selectively quantified after removing the TMRE florescence in cell bodies using a customized script. As a result, we found that both the total TMRE fluorescence intensity in the neurite compartment and the number of detected mitochondria were decreased by 59% and 58%, respectively, in cells treated with 10 nM BTZ compared with the vehicle-treated condition (Fig. 2A,B).

As a decrease in TMRE staining can indicate either a loss of mitochondrial membrane polarity or a decrease in overall mitochondria number, we next quantified mitochondrial number using a membrane potential-independent mitochondrial marker ATP5β. Treatment with 10 nM BTZ led to a 32% decrease in ATP5β-positive puncta in the neurite compartment (Fig. 2C,D). Given that the decrease in TMRE staining following BTZ treatment was nearly twice the magnitude of the decrease in ATP5β-positive mitochondria, it is likely that BTZ has a combined effect of decreasing mitochondrial number and inducing mitochondrial depolarization.

We next examined the effects of BTZ on mitochondrial function and cellular energetics. We measured the levels of cellular ATP using a luminescence-based ATP assay (Fig. 2E). A dose-dependent decrease in ATP was observed at 24, 48 and 72 h after BTZ treatment. At 24 h, the difference between doses of BTZ was moderate, with 5 nM, 10 nM and 15 nM BTZ decreasing ATP levels to 91.3%, 70.0% and 61.8% relative to those of controls, respectively. However, at 72 h, the difference between these doses was more striking with ATP levels of 93.6%, 36.7% and 17.5% relative to those of controls, respectively. To confirm that the observed decrease in ATP levels was the result of a decrease in mitochondrial respiration, we directly measured oxygen consumption using a Seahorse XF Analyzer. hiMNs treated with 10 nM BTZ for 24 h displayed a decrease in both basal (95% c.i., −48.45 to −31.05) and maximal (95% c.i., −62.68 to −17.19) respiratory capacity, as indicated by oxygen consumption in the absence and presence of the decoupling agent FCCP, respectively (Fig. 2F-H). Importantly, this timepoint (24 h) is before neurite degeneration occurs, so the effect on oxygen consumption cannot be explained by a loss of cellular mass.

An additional indicator of mitochondrial function is the presence of reactive oxygen species (ROS). When the electron transport chain is compromised, ROS are generated, and this can lead to oxidative damage to proteins and DNA. Increased ROS are observed in dorsal root ganglion neurons in rodent models of BIPN (Dong et al., 2021). Using a luciferase assay to detect ROS, we observed a dose-dependent increase in ROS in hiMNs treated with BTZ (Fig. S2A). The effect began after 24 h of BTZ treatment and was far more dramatic in the 10 nM BTZ and 15 nm BTZ conditions than at 5 nM. The increase in ROS was inversely proportional to the decrease in ATP observed, suggesting that the two processes might share a common mechanism (Fig. S2B).

### Caspase activation is not necessary for BTZ-induced degeneration in hiMNs

**Fig. 3.**
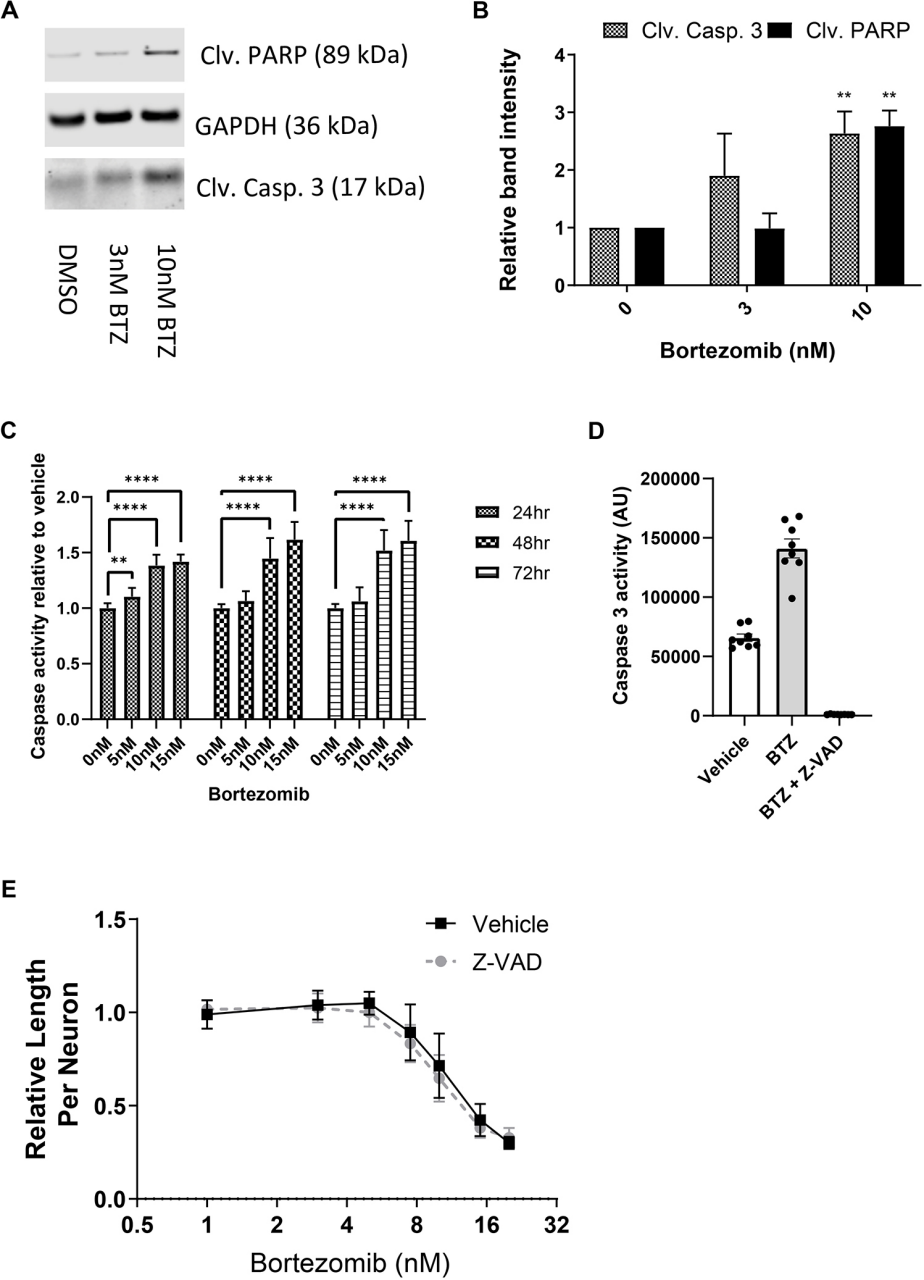
**Caspase activation is not necessary for bortezomib-induced neurite degeneration in hiMN.** (A) Representative western blots for cleaved caspase-3 and cleaved PARP in hiMNs treated with vehicle, 3 nM BTZ or 10 nM BTZ for 72 h. (B) Densitometry quantification of western blots in A. Data were tested with two-tailed unpaired Student's *t*-test. **P<0.005 (*n*=3). (C) Caspase-3 activity as measured by luminescence-based assay at 24, 48 or 72 h after BTZ treatment. Data were tested with a one-way ANOVA with Dunnett's post-hoc test. **P<0.005; ****P<0.00005 (*n*=16). (D) Caspase-3 activity in hiMNs pre-treated for 2 h with either vehicle or 50 μM Z-VAD-FMK before the addition of 10 nM BTZ for 72 h. AU, arbitrary units. (E) hiMNs were treated with vehicle or 30 μM Z-VAD-FMK for 2 h prior to the addition of varying doses of BTZ. Neurite length was quantified at 72 h using automated neurite tracing software. Data were analyzed by two-way ANOVA with Dunnett's multiple-comparisons test (*n*>18). All error bars represent s.e.m.

Some forms of axon degeneration, mitochondrial dysfunction and mitochondrial depolarization have been linked to the activation of caspases through the release of pro-apoptotic factors from the mitochondrial matrix (Cosker et al., 2013; Geisler et al., 2019a,b; Schoenmann et al., 2010). To determine whether caspase activation plays a role in the degeneration of BTZ-treated hiMNs, we performed western blotting for cleaved caspase-3 and cleaved PARP 24 h after BTZ exposure. Treatment with 10 nM BTZ caused an increase in both cleaved caspase-3 and cleaved PARP (Fig. 3A,B). Although 3 nM BTZ showed a trend towards increased caspase activation, the change was not significant. The increase in cleaved caspase-3 at higher doses of BTZ corresponded with a progressive increase in caspase-3 activity, as measured by a luminescence-based caspase activity assay (Fig. 3C). Interestingly, caspase activation occurred rapidly after BTZ treatment and preceded any detectable change in neurite length. Similar to our observations for ATP loss and ROS production, caspase activity was only significantly induced at those concentrations of BTZ sufficient to induce axon degeneration (greater than or equal to 10 nM), whereas lower doses of BTZ that induced growth arrest showed no increase in caspase activity relative to vehicle-treated cells.

It has been proposed that BTZ can induce axon degeneration through the disruption of trophic factor signaling (Youk et al., 2017). Axon degeneration following trophic factor withdrawal has been shown to rely on the activation of executioner caspases 3 and 6 (Simon et al., 2012). However, other mouse models have suggested that caspase activation following BTZ treatment is not required for axon degeneration (Geisler et al., 2019a). To determine whether caspase activity is required for BTZ-induced axon degeneration in human neurons, we used a small peptide pan-caspase inhibitor, Z-VAD-FMK. When hiMNs were pre-treated with 10 μM Z-VAD-FMK for 2 h prior to the addition of BTZ, caspase-3 activity was now indistinguishable from that of untreated neurons, essentially absent (Fig. 3D). Despite being sufficient to completely reverse the effects of BTZ on caspase-3 activity, the pre-treatment with Z-VAD-FMK had no effect on the BTZ-induced reduction in neurite outgrowth (Fig. 3E), and we therefore conclude that the neuropathy induced by BTZ in human neurons is independent of caspase activity. As we did not detect any cell death even at high concentrations of BTZ, the caspase activation it induces appears not to be neurotoxic.

### BTZ-induced degeneration is NAD dependent in hiPSC-derived neurons

**Fig. 4.**
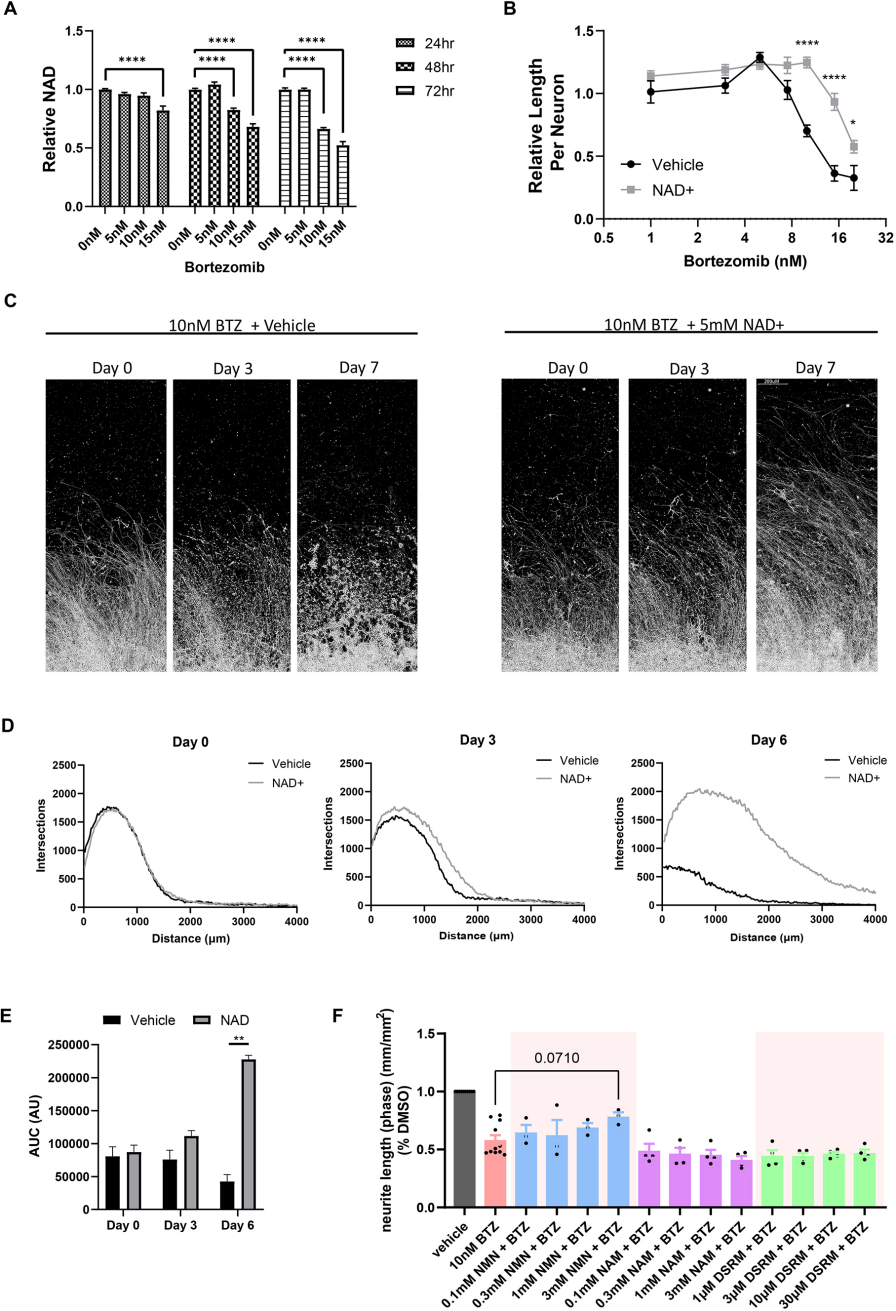
**Bortezomib-induced neurite degeneration in hiMNs is dependent on NAD depletion.** (A) NAD measurements in hiMNs treated with varying doses of BTZ for 24, 48 or 72 h. Data were tested with a one-way ANOVA. Significant effects were observed for BTZ treatment; *F* (3, 60)=11.45, *P*<0.0001; post hoc Dunnett's multiple-comparisons test. ****P<0.0005 (*n*=16). (B) hiMNs treated with 5 mM NAD or vehicle for 2 h prior to the addition of varying doses of BTZ. Neurons were fixed after 72 h and neurite length per neuron measured. Data were analyzed by two-way ANOVA. Significant effects were observed for NAD; treatment *F* (1, 239)=53.08, *P*<0.0001; time *F* (7, 239)=40.58, *P*<0.0001; and interaction *F* (7, 239)=7.246; post hoc Sidak's multiple-comparisons test; *P<0.05; ****P<0.00005. (C) Representative images of hiMN spot cultures at 0, 3 and 7 days after treatment with 10 nM BTZ either in the presence or absence of 5 mM NAD. Scale bar: 200 μm. (D) Representative Sholl analysis of spot cultures from C measured at 0, 3 and 6 days. (E) AUC quantification of axon density in spot cultures. Values represent the average and s.e.m. for three biological replicates 0, 3 and 6 days after BTZ treatment. Data were tested with a two-way ANOVA. Significant effects were observed for treatment *F* (1, 4)=35.3, *P*<0.004; **P<0.01 by post hoc Sidak's multiple-comparisons test. (F) Quantification of the neurite length (mm/mm2) in NAM- (*n*=4), NMN- (*n*=3) and DSRM-3716- (*n*=4) treated conditions. Statistical measurements were done with one-way ANOVA with Dunnett's post hoc test. All error bars represent s.e.m.

The axon fragmentation that is a key feature of Wallerian degeneration is caspase-independent (Finn et al., 2000). In Wallerian degeneration, activation of SARM1 and a loss of axonal NMNAT2 contribute to a rapid depletion of axonal NAD (Gerdts et al., 2013; 2015). In rodent models of BIPN, a decrease in NAD is observed before any detectable axon fragmentation takes place (Geisler et al., 2019a). To determine whether a similar Wallerian-like NAD^+^ depletion process might play a role in BTZ-induced degeneration of human neurons, we examined NAD levels in hiMNs treated with BTZ using a luciferase detection assay (Fig. 4A). After 24 h of BTZ treatment, there was a significant decrease in cellular NAD for 15 nM BTZ-treated cells (95% c.i., −0.1006 to −0.2567), but not in the 5 nM or 10 nM conditions. However, at 48 h, there was a significant decrease in NAD levels in both the 10 nM and 15 nM exposure groups. Conversely, there was no change in NAD levels in hiMNs treated with 5 nM BTZ at any timepoint, consistent with the fact that this concentration of BTZ does not cause axon degeneration.

Previous studies of Wallerian degeneration in transected mouse neurons have shown that increasing intracellular NAD by a variety of mechanisms can delay the onset of the Wallerian degeneration, demonstrating that NAD depletion is a causal event in Wallerian axon degeneration and not a downstream effect of the degenerative process. We therefore tested whether the same was true in BTZ-treated hiMNs by treating the hiMNs with exogenous 5 mM NAD starting 2 h prior to BTZ exposure. Treatment with NAD had no effect on neurite length in the absence of BTZ (Fig. 4A,B). However, at concentrations of BTZ greater than 10 nM, NAD pre-treatment provided a significant protective effect relative to vehicle-treated controls. The effect was greatest at 15 nM BTZ (95% c.i., +33.6 to +80.4). To confirm that exogenous NAD prevented axon degeneration and did not just increase neurite growth, we examined the effects of NAD pretreatment using longitudinal live-cell imaging. Spot cultures treated with 15 nM BTZ normally undergo rapid degeneration after 24 h of exposure and with a total loss of axons around 5 days. Pretreatment with 5 mM NAD completely prevented any sign of axon degeneration after 5 days of BTZ exposure to the spot cultures and promoted further neurite growth beyond that observed at the time of BTZ addition (Fig. 4C-E; Fig. S3).

Having identified NAD^+^ as an important mediator of BTZ-induced toxicity in human neurons, we set out to better understand the molecular pathways linking NAD^+^ to BTZ-induced degeneration, specifically whether the observed degeneration was mediated by a similar signaling pathway as that described in Wallerian degeneration (Chang et al., 2016). Several compounds previously demonstrated to inhibit Wallerian degeneration were tested for their effects on BTZ-induced axon degeneration in hiMNs. All compounds were added 2 h prior to BTZ treatment. We observed a trend towards increasing neurite length when hiMN were pre-treated with increasing doses of nicotinamide mononucleotide (NMN), a direct precursor to NAD. However, at the highest dose tested (3 mM), NMN only produced a 34% decrease in BTZ toxicity (Dunnett's test, adjusted *P*=0.071) (Fig. 4F). Nicotinamide (NAM), a more upstream precursor of NAD, had no effect on BTZ-induced axonal degeneration at concentrations up to 3 mM (Fig. 4F). Finally, unexpectedly, the small-molecule SARM1 inhibitor DSRM-3716 had no effect on BTZ-induced axon toxicity in hiMNs at concentrations up to 30 μM, which are beyond those concentrations previously shown to block SARM1-mediated axon degeneration (Hughes et al., 2021) (Fig. 4F).

**Fig. 5.**
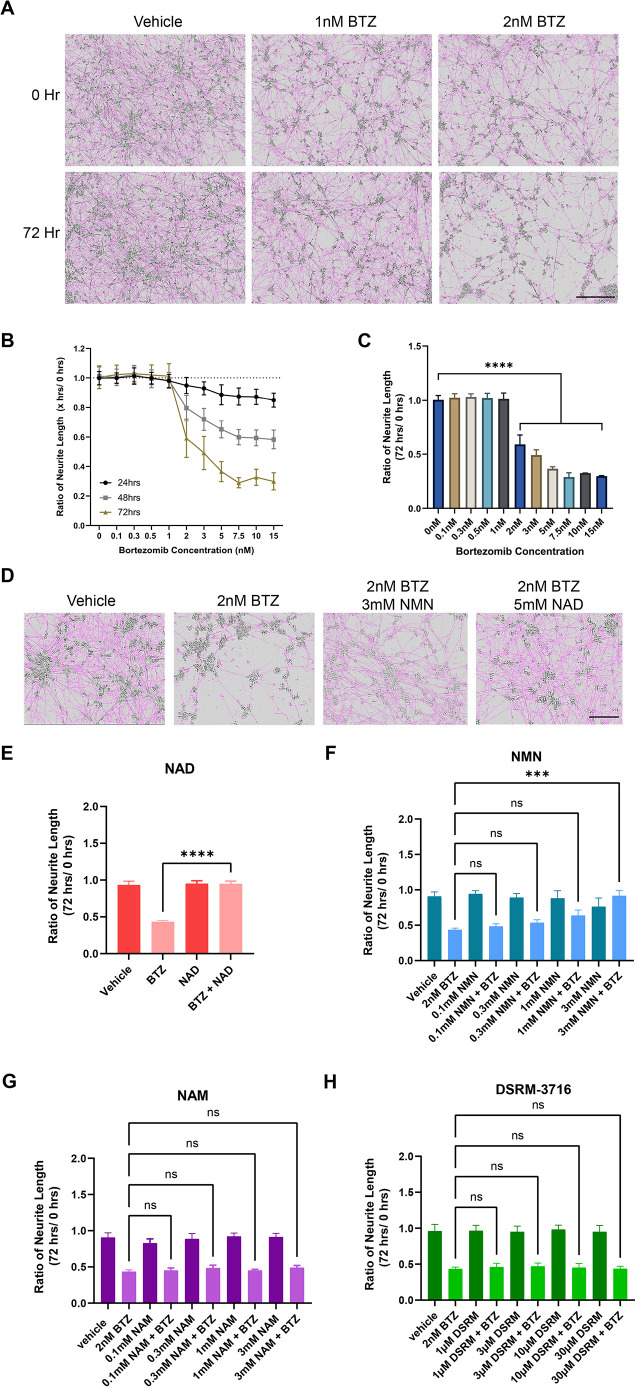
**Bortezomib-induced degeneration is NAD and NMN dependent in human sensory neurons.** (A) Representative images of cells at 0 and 72 h in DMSO-treated and in 1 nM (non-degenerative) and 2 nM (degenerative) BTZ-treated conditions. Scale bar: 400 µm. (B) Graph showing the dose response effects of BTZ on axon degeneration at 24, 48 and 72 h post treatment. (C) Quantification of BTZ-induced neurodegeneration at 72 h post treatment (one-way ANOVA with Dunnett's post hoc test; ****P<0.0001; two to five independent experiments; five to ten wells/condition in each experiment). (D) Representative images of sensory neurons treated with DMSO, 2 nM BTZ or 2 nM BTZ together with 5 mM NAD or with 3 mM NMN at 72 h post treatment. Scale bar: 400 µm. (E) Graph showing that treatment with NAD^+^ (5 mM) prevents BTZ-induced degeneration (one-way ANOVA with Dunnett's post hoc test; ****P<0.0001; *F*=45.95; three independent experiments; ten wells per condition). (F) Treatment with NMN (1 mM and 3 mM) rescues BTZ-induced degeneration (one-way ANOVA with Dunnett's post hoc test; ns, not significant; ***P<0.001; *F*=8.142; three independent experiments; ten wells per condition). (G) Treatment with NAM (0.1-3 mM) does not rescue BTZ-induced degeneration (one-way ANOVA with Dunnett's post hoc test; *P*<0.001; *F*=24.04; three independent experiments; ten wells per condition). (H) Treatment with DSRM-3716 (0.1-3 mM) does not rescue BTZ-induced degeneration (one-way ANOVA with Dunnett's post hoc test; *P*<0.001; *F*=16.53; three independent experiments; ten wells per condition).

Although BTZ is associated with rare cases of motor neuropathy, sensory neuropathy is a far more common adverse event associated with the drug. To determine whether the disease model described was also generalizable to sensory neurons, we tested the ability of exogenous NAD to protect hiPSC-derived sensory neurons (hiSNs) from BTZ toxicity. hiSNs were treated with varying concentrations of BTZ (0.1-15 nM) at 28 days *in vitro* and imaged over 72 h using a live imaging, neurite-tracking system (Incucyte, Essen Bioscience). Neurodegeneration was quantified as reduction in the number of neurites over time. All doses of BTZ higher than 2 nM (average ratio of neurite number at 72 h/0 h=0.59) caused axon degeneration by 72 h post treatment, with 15 nM being the most severe (average ratio of neurite number at 72 h/0 h=0.29) (one-way ANOVA with Dunnett's post hoc test, *P*<0.0001) (Fig. 5A-C). Based on these results, we chose to use 2 nM as the lowest dose causing consistent axon degeneration at 72 h post treatment in hiPSC-derived sensory neurons for the experiments.

We then examined whether those molecules shown to be protective in our motor neuron model were similarly protective in hiSNs. Pretreatment of hiSNs with NAD (5 mM) for 2 h prior to BTZ treatment, similarly to hiMNs, completely rescued BTZ-induced neurodegeneration in the human sensory neurons (ratio of neurite number at 72 h/0 h=0.95) (Fig. 5D,E).

In contrast, treatment with NMN, which trended towards some protection in the hiMN model, produced a substantial rescue from BTZ-induced axonal degeneration at 1 mM (ratio of neurite number at 72 h/0 h=0.64) and 3 mM (ratio of neurite number at 72 h/0 h=0.92) in hiSNs (Fig. 5F). The greater effect in sensory neurons than in motor neurons might reflect either cell type-specific differential sensitivity or increased uptake of NMN. However, exactly mirroring the findings observed in hiMNs, both NAM and DSRM-3716 had no effect on BTZ-induced axonal degeneration at concentrations up to 3 mM and 30 μM, respectively (Fig. 5G,H), despite SARM1 being highly expressed in hiSNs (Fig. S5).

## DISCUSSION

CIPN has proved to be an intractable hurdle in the treatment of multiple forms of cancer owing to a lack of any efficacious therapies to either prevent or reverse the nerve damage caused by chemotherapeutics. Two primary problems in the development of therapeutics for the treatment of CIPN have been a lack of targets for therapeutic intervention owing to an incomplete mechanistic understanding of the disease and a failure of those treatments found to be efficacious in preclinical animal models to demonstrate any clinical benefit in humans. Development of humanized models for CIPN might address both issues by providing insight into the mechanism of chemotherapy-induced nerve injury in human neurons and by allowing for screening for novel therapeutics.

It has previously been reported that hiPSC-derived neurons treated with BTZ display a decrease in neurite length (Wheeler et al., 2015; Wing et al., 2017). We now provide further characterization of this phenomenon and developed methods specifically designed to determine whether the observed decrease in neurite length represents a dying-back degeneration of neuronal processes, as seen in CIPN, as opposed to a slowing of neurite extension. First, we show that loss of neurites occurs without a loss of cell bodies, consistent with evidence that CIPN is primarily an axonopathy (Quasthoff and Hartung, 2002). Second, our spot culture-based live-imaging system provides definitive evidence that degeneration proceeds in the distal-to-proximal manner characteristic of peripheral neuropathies, including various forms of CIPN (Liu et al., 2018). Third, the delayed nature of the degeneration closely matches the time course of BTZ-induced degeneration observed in rodent models of BIPN (Chaudhry et al., 2008). These similarities between our *in vitro* human neuronal model and *in vivo* rodent studies support our conclusion that human neurons in culture can recapitulate the degenerative process responsible for BIPN.

We also demonstrate similarities between hiPSC-derived neurons and rodent models of BIPN at the molecular level. Through a combination of imaging and biochemical studies, we reveal that BTZ-induced degeneration is dependent on the depletion of cellular NAD^+^. NAD^+^ was first implicated in axonal degeneration through study of the Wallerian degeneration slow mouse (Wld^S^), in which the abnormal targeting of an NAD^+^-producing enzyme to the axon led to elevated levels of axonal NAD^+^ and axonal protection following nerve transection (Lunn et al., 1989; Mack et al., 2001). It is now appreciated that NAD^+^ acts downstream of a variety of insults and represents the key determinant between axon degeneration and axon maintenance (Gerdts et al., 2016). Prior studies have demonstrated that NAD^+^ loss is also necessary for vincristine- and BTZ-induced axon degeneration in mice, associating traumatic and chemotherapy-induced neuropathy with a common mechanism (Geisler et al., 2016, 2019a). Our findings therefore provide cross-species validation for the potential utility of therapeutics targeting NAD^+^ metabolism in the treatment of BIPN.

It is unlikely, however, that direct supplementation of NAD^+^, as used in this study, will be an effective therapeutic strategy, given that it is poorly cell permeant. We were able to overcome this limitation in our *in vitro* system with application of sufficiently high concentrations of NAD^+^, but the millimolar extracellular concentrations necessary to provide protection would be very difficult to achieve in patients. One potential way to overcome this challenge would be to use uncharged precursors to NAD^+^ that would have improved bioavailability. Two such precursors, NMN and NAM, are already widely available as dietary supplements. Our study suggests that NMN has a similar protective effect as NAD^+^, although the concentrations necessary to achieve a protective effect were only modestly lower than the concentration of NAD^+^. To our surprise, NAM was not protective. One possible explanation of this observation is that NAM is further upstream than NMN in the NAD^+^ salvage pathway and, thus, might require longer pre-treatment intervals or higher doses than we examined here.

An alternative explanation could be that at the concentrations used, NAD^+^ and NMN have an off-target effect on a cellular process not normally dependent on NAD^+^ signaling. However, numerous prior studies of axonal degeneration following axon transection, the prototypical model for Wallerian degeneration, required similar millimolar concentrations of NAD and NMN to demonstrate a protective effect at these high exposure levels (Wang et al., 2005; Sasaki et al., 2006; Wang et al., 2015). Additionally, the finding that NAD^+^ is depleted in neurons after BTZ treatment lends further support to the postulate that NAD^+^ is the endogenous signaling mediator that rescues axons from the chemotherapy-induced degeneration. Therefore, although we are unable to definitively rule out the possibility that both NAD^+^ and NMN might be acting on an unidentified pathway unrelated to NAD^+^ metabolism, we feel this is unlikely to be the case.

Another unexpected finding was that the small molecule SARM1 inhibitor DSRM-3716 was not protective, given the well-established link between SARM1 activation, NAD^+^ depletion, and axon degeneration despite the key regulators of thesis pathway, NMNAT2 and SARM1, being highly expressed in iSNs (Deng et al., 2022 preprint). Furthermore, previous studies have demonstrated that SARM1 is necessary for vincristine- and BTZ-induced axon degeneration in mice and vincristine-induced degeneration in hiPSC derived neurons (Chen et al., 2021; Geisler et al., 2016, 2019a,b). However, these studies utilized SARM1 knockout lines rather than chemical SARM1 inhibitors, raising the possibility that a greater degree of SARM1 inhibition than achievable with current small molecule inhibitors is necessary to prevent BTZ induced axon degeneration in human neurons. Less likely, although possible, is that BTZ might act through a SARM1-independent mechanism in human but not mouse neurons. Further studies using SARM1 knockout hiPSCs will be necessary to distinguish between these two scenarios and determine whether SARM1 is a potential target for the prevention of BIPN.

Although our findings and those of others suggest that BTZ induces axon degeneration through depletion of axonal NAD^+^, the steps upstream of the loss of NAD^+^ are still to be determined. It has been proposed that mitochondrial dysfunction is a key mediator of the neurotoxicity of BTZ. There are an increased number of swollen, vacuolar mitochondria in the nerves of rats treated with BTZ, and treatment of mice with the mitoprotective agent acetyl-L-carnitine decreases BTZ-induced hyperalgesia (Zheng et al., 2012). BTZ also causes a loss of mitochondrial polarization in the axons of primary rodent neurons (Geisler et al., 2019a). Moreover, in hiPSC-derived sensory neurons, BTZ decreases mitochondrial mobility and increases proteins associated with mitotoxicity (Hrstka et al., 2021). Our findings also suggest that mitochondrial dysfunction likely plays a key role in human neurons as well. A loss of mitochondrial membrane potential, ATP depletion and ROS production are, we find, all early events following BTZ treatment in hiPSC-derived neurons. In our assays, these processes all precede any detectable decrease in NAD or axonal degeneration. The possibility that mitotoxicity is an early mediator of BTZ-induced axon degeneration is intriguing, as direct mitochondrial poisons initiate axon degeneration through a SARM1-NAD^+^-dependent mechanism (Loreto et al., 2020). However, without a more complete understanding of the mechanism by which BTZ causes mitochondrial dysfunction, it is not yet possible to identify conclusively whether mitochondrial toxicity is the causative triggering insult leading to axon degeneration through NAD^+^ depletion.

Although several molecular mechanisms by which BTZ induces axon degeneration in human neurons are revealed by our study – especially NAD depletion – there are still gaps. Some mechanisms of BTZ neurotoxicity implicated in rodent models have yet to be confirmed in human neurons, including alterations in NF-κB signaling, slowing of axonal trafficking via stabilization of axonal microtubules, and overactivity of the TRPA1 channel (Alé et al., 2014; 2016; Staff et al., 2013; Trevisan et al., 2013; Wang et al., 2011). One area in which we find that human neuronal and rodent models are discrepant is in the role of caspases in BTZ-induced neurotoxicity. Caspases are protective against BTZ-induced degeneration in mouse models of BIPN, which has led to the hypothesis that mitochondrial dysfunction activates caspases, which in turn activate SARM1 (Geisler et al., 2019a). Although we did observe a significant increase in caspase activity following BTZ exposure, small-molecule caspase inhibitors did not have any protective effect on human neurons treated with BTZ. This finding suggests that caspases might not be an effective target for the treatment of BIPN and highlights the importance of having humanized model systems for the study of BIPN to validate therapeutic candidates.

The merits and pitfalls of *in vitro* models for CIPN have been discussed elsewhere (Lehmann et al., 2020), but some points with specific implications in our study warrant reiteration. A major consideration of any study using hiPSC-derived cells is the extent to which the generated cells resemble the primary tissue being studied. The reason why sensory neurons are most susceptible to BTZ toxicity *in vivo* is not known. One hypothesis is that the peripherally located cell bodies of sensory neurons are exposed to much higher levels of BTZ than the cell bodies of motor neurons, which lie behind the blood-brain barrier. This hypothesis is supported by the finding that when BTZ is applied selectively to the axons of primary mouse neurons, it has less toxicity than when the cell bodies are exposed (Geisler et al., 2019a). BIPN is predominantly a disease of sensory fibers, the results obtained in this study show that hiPSC-derived motor neurons were vulnerable as well. Interestingly, BTZ is associated with motor and autonomic neuropathies, although it is rare, suggesting a general neurotoxic action (Grammatico et al., 2016). Moreover, previous studies have demonstrated BTZ-associated neurotoxicity in a variety of neuronal subtypes *in vitro*, bolstering the argument that BTZ toxicity is not specific to sensory neurons (Wheeler et al., 2015; Wing et al., 2017). Lastly, we demonstrated that those molecules identified as protective against BTZ-induced neuronal degeneration in hiMNs had a similar protective effect in hiSNs.

One of the greatest advantages of a model for BIPN using hiPSC-derived neurons is the ability to scale assays to perform large screens, something not feasible using primary neurons. Unbiased screens for targets that prevent BTZ-induced axon degeneration will likely both deepen our understanding of chemotherapy-induced neuropathy and provide new paths to treat it. In summary, we have developed a variety of assays utilizing hiPSC-derived neurons to model BTZ-induced neurotoxicity and employed these assays to identify NAD metabolism as a key mediator of BTZ-induced axonal degeneration in human sensory and motor neurons. We show that exogenous NAD is capable of completely blocking the degenerative effects of BTZ treatment. We also reveal a potential role for mitochondrial dysfunction in the pathogenesis of BTZ-induced degeneration in human neurons. It will now be important to clarify the molecular mechanisms by which BTZ disrupts mitochondria and induces NAD depletion, and to explore which interventions are most neuroprotective without interfering with the antimitotic activity of the cancer therapies.

## MATERIALS AND METHODS

### Compounds

Nicotinamide (NAM) and nicotinamide mononucleotide (NMN) were obtained from Sigma-Aldrich (N3376 and N3501, respectively), DSRM-3716 and BTZ were obtained from Tocris (7414 and 7282, respectively).

### hiPSC lines

hiMNs were differentiated from the SAH0049 line (Sahin laboratory, Boston Children's Hospital). SAH0049 was derived from fibroblasts donated by a 45-year-old healthy female. hiSNs for neurite length experiments were differentiated from the LiPSC-GR1.1 line derived from cord blood of a male donor (https://commonfund.nih.gov/stemcells/lines). hiSNs for SARM1 expression measurement were differentiated from the National Institutes of Health (NIH)-approved embryonic stem cell line WA09 (WiCell Research Institute).

### hiPSC culture

hiPSCs were maintained on Geltrex LDEV-free matrix (Themo Fisher Scientific) in StemFlex (Thermo Fisher Scientific). Cells were passaged at a ratio of 1:12 every 4-6 days, when they were at ∼80% confluence, using ReLeSR (Stem Cell Technologies) enzyme-free passaging reagent. After ten passages, a new vial of hiPSCs were thawed or the established culture karyotyped. The hiPSC line SAH-0047 was provided by Mustafa Sahin’s laboratory, Boston Children's Hospital, Boston, MA, USA.

### Motor neuron differentiation

hiPSCs were differentiated into hiMNs as previously described (Wainger et al., 2014). Briefly, hiPSCs were split to single cells using Accutase (Stem Cell Technologies) and plated at a density of 5×10^5^ cells/ml in a 10 cm Geltrex-coated plate in StemFlex medium with 1 µM Y-27632 (Tocris, 1254). After the first day, cells were maintained in motor neuron medium, a 50/50 mixture of Dulbecco's Modified Eagle Medium (DMEM)/F12 (Thermo Fisher Scientific) and neurobasal medium (Thermo Fisher Scientific) supplemented with B27 (Thermo Fisher Scientific), N2 (Thermo Fisher Scientific), GlutaMAX (Thermo Fisher Scientific) and non-essential amino acids (Corning) as per the manufacturer's recommendations. For the first five days, the motor neuron medium was supplemented with SB431542 (10 μM, Cayman Chemical, 13031) and LDN (100 nM, Sigma-Aldrich, SML0559-5MG) to neuralize the cells, and retinoic acid (1 μM, Sigma-Aldrich, R2625) and SAG (1 μM, Tocris, 4366) to posteriorize and ventralize the cells. On the sixth day, SB431542 and LDN were replaced with SU5402 (4 μM, Sigma-Aldrich, SML0443-5MG) and DAPT (5 μM, Tocris, 2634) to specify motor neuron identity. After an additional 10 days, the cells were harvested by incubation with Accutase at 37°C for 45 min. The detached cells were washed twice with DMSO and filtered through a 70 μm cell strainer. A small number of cells were plated and stained for the lineage markers MAP2 (1:2000, Millipore, AB15452) and ISL1 (1:1000, Abcam, ab279600) to assess purity. The remainder of cells were frozen in a 1:1 mixture of motor neuron medium and Cryostor CS10 (Stem Cell Technologies) in liquid nitrogen for future use.

Before each assay, hiMNs were thawed and sorted for CD56 (NCAM1) expression. The thawed cells were resuspended at 1×10^8^ cells/ml in MACS buffer (0.5% bovine serum albumin and 1 mM EDTA in PBS) with PE-conjugated anti-CD56 antibody (1:10, BD Biosciences, 555516). Cells were incubated with the antibody on ice for 30 min before washing once with MACS buffer and resuspending in MACS buffer with anti-R-phycoerythrin magnetic particles (1:10, BD Biosciences, 557899), followed by incubation at room temperature for 45 min. Bead-cell conjugates were captured by flowing the cell suspension through large cell MACS columns (Miltenyi Biotec, 130-042-202). After elution from the columns, cells were counted and plated in motor neuron medium supplemented with growth factors [35 μg/ml ascorbic acid (Sigma-Aldrich, A4403), 10 ng/ml recombinant human BDNF (Thermo Fisher Scientific, PHC7074), 10 ng/ml recombinant human GDNF (Thermo Fisher Scientific, PHC7045) and 10 ng/ml recombinant human CNTF (Thermo Fisher Scientific, PHC7015)] on plates coated with poly-D-lysine and laminin at a density appropriate for the assay.

### Dissociated neurite length assay

hiMNs were plated at a density of 2000 cells per well in 384-well plates. Compounds were diluted in culture medium and added either 24, 48 or 72 h after plating as required for the assay. Cells were fixed with 4% paraformaldehyde for 25 min at room temperature either 24 or 72 h after chemotherapy addition. Fixed cells were then blocked in blocking buffer [1% blocking reagent (Sigma-Aldrich, 11096176001) with 0.1% Triton X-100 in PBS] for 30 min and stained overnight at 4°C with anti-βIII-tubulin (Sigma-Aldrich, T8660) diluted 1:1000 in blocking buffer. The secondary antibody (Alexa Fluor 488 goat anti-mouse, 1:10,000, Invitrogen, A-11001) was diluted in blocking buffer and incubated for 2 h at room temperature. Images were captured and analyzed with the XTi Arrayscan high content imager (Thermo Fisher Scientific), using the neuronal profiling plugin for neurite quantification (https://assets.thermofisher.com/TFS-Assets/BID/brochures/ArrayScanXTI_HCAReader.pdf). Briefly, the software takes two-channel images, one channel for DAPI-labeled nuclei and the other for neuron cell bodies and neurites labeled with βIII-tubulin. The DAPI channel is used to segment the βIII-tubulin channel into individual neurons. Then, neurites are automatically traced from the βIII-tubulin-positive cell bodies using a combination of signal intensity over background, width and directionality (Fig. S4).

### Western blotting

Proteins from ∼1×10^6^ hiMNs were harvested in 50 μl lysis buffer (50 mM Tris-HCl pH 7.4, 100 mM NaCl, 1 mM EDTA, 0.5% NP-40 and 10% glycerol) for each sample. Relative protein concentration for samples within an experiment was determined by Bradford assay and samples were diluted accordingly with lysis buffer to normalize protein concentrations. Lysates were separated on 4-12% Bis-Tris SDS-PAGE gels and blotted with rabbit anti-cleaved caspase-3 (1:1000, Cell Signaling Technology, 9664) or rabbit anti-cleaved PARP (Cell Signaling Technology, 5625) overnight at 4°C. Anti-GAPDH (1:2000, Abcam, ab8245) was blotted on every gel for normalization. LI-COR fluorescent secondary antibodies (1:10,000, LI-COR, 926-68070 and 926-32211) were used to probe bands. Gels were imaged and bands quantified on the LI-COR Odyssey CLX imager.

### Luminescence assays

hiMNs were plated at a density of 10,000 cells per well in a 384-well format and treated with chemotherapy 3 days later. Cellular ATP, ROS, NAD^+^ or caspase activity were assayed at 24 and 72 h using Promega CellTiter Glo, ROS Glo, NAD Glo or Caspase Glo assays, respectively. For CellTiter Glo, ROS Glo and NAD Glo assays, treatment with 50 µM menadione (Sigma-Aldrich, M5625-25G) for 6 h prior to the assay was used as a positive control. For Caspase Glo, no positive control was necessary given the dramatic increase in caspase activity after BTZ treatment.

### Seahorse assay

hiMNs were plated at a density of 100,000 cells per well in a poly-D-lysine- and laminin-coated Agilent Seahorse XFe24 assay plate. After 4 days, the cells were treated for 24 h with vehicle or 15 nM BTZ. Mitochondrial respiration was then quantified 24 and 72 h after chemotherapy addition as previously described for primary mouse neurons (Ribeiro et al., 2015). Briefly, oxygen consumption was read three times 3 min apart for each of four conditions: (1) baseline, (2) 1 µM of the ATP synthase inhibitor oligomycin (Agilent Seahorse XF Cell Mito Stress Test Kit, 103010-100) to measure oxidative phosphorylation, (3) 1 µM of the uncoupler FCCP (Agilent Seahorse XF Cell Mito Stress Test Kit, 103010-100) to measure maximal respiratory capacity and (4) 1 µM of the complex I inhibitor rotenone (Agilent Seahorse XF Cell Mito Stress Test Kit, 103010-100) to proton leak. Data were analyzed using the Agilent Seahorse analysis software. Averages and errors were calculated from five or six biological replicates per condition.

### Spot cultures

hiMNs were resuspended at a density of 16,000 cells/μl in growth medium supplemented with growth factors. 6 μl spots were placed in the center of each well in a completely dry poly-D-lysine- and laminin-coated glass-bottomed six-well plate (MatTek, P06G-1.5-20-F) and incubated in a humidified 37°C degree incubator for 45 min. Growth medium was then added stepwise around the spot culture to fill the well, with care being taken not to disrupt the cell bodies. Compounds were diluted in culture medium and added 72 h after plating.

Imaging of spot cultures was performed in a CO_2_- and temperature-controlled incubator using phase contrast at 10× magnification on a Nikon TI Eclipse inverted microscope. Images were acquired just prior to chemotherapy addition and 24, 48, 120 and 168 h thereafter. In each case, an image encompassing the entire cell body spot and neurite halo was constructed using the optimal patch stitching algorithm in NIS Elements (Nikon) with 10% overlap. If at any time point it was not possible to obtain a clear, unobstructed image of the spot due to artifacts or disruption of the culture, all data from that culture were excluded from analysis. Images were then imported to ImageJ where rolling ball background subtraction, contrast enhancement and image thresholding were performed to create binary masks of images. Finally, the cell body region was manually defined and Sholl analysis performed using a Fiji macro gifted by L.C. For a detailed description of the method of Sholl analysis and the base code for the Fiji macro, see Ferreira et al. (2014). Briefly, the macro takes as input a binarized image in which axons are white and background black. The user then manually defines the cell body region and the macro uses this outline to generate concentric, 1-pixel-wide rings at defined distances. The number of contiguous white regions on this ring (representing the points at which an axon intersects it) are then counted in an automated manner. A ring size of 60 pixels was used for the data presented.

### Live-cell mitochondrial imaging

On day 14 post differentiation, sorted hiMNs were seeded with approximately 1×10^4^ neurons per well in 96-well plates. On day 17, the cells were treated with 10 nM BTZ. To measure mitochondrial membrane potential, live-cell imaging was done on day 20 after incubation with 5 nM TMRE dye (I34361, Thermo Fisher Scientific) for 20 min. After washing with PBS, the cells were imaged using ArrayScan IXM imaging platform (Molecular Devices, San Jose, CA, USA) with a 10× objective with 2×2 binning. The images were captured from at least five wells per field and four fields per condition. The TMRE fluorescence was quantified using a customized script from metaXpress software (Molecular Devices).

### Fixed-cell mitochondrial imaging

To measure axonal mitochondria number, the cells were seeded with approximately 1×10^4^ neurons per well in 96-well plates, fixed and labeled with a mitochondrial marker, anti-ATP5β antibody (1:500, ab14730, Abcam) and anti-βIII-tubulin antibody (1:1000, T8660, Sigma-Aldrich). The cells were then imaged with TiEclipse microscopy (Nikon) using Plan Fluor 60× objective, NA 0.70. The 100 mm axonal length was measured and mitochondria number quantified using ImageJ.

### Incucyte analysis

The approximately 2×10^3^ neurons were seeded in 384-well plates at cells per well. Plates were imaged in Incucyte S3 live cell imaging incubator (Essen BioScience, Ann Arbor, MI, USA) before and after compound treatment. Ten wells per condition were captured. The neurite outgrowth was measured using Incucyte analysis pipeline (Essen BioScience). To analyze the neurite growth, the 96 h treatment conditions were normalized with pre-treated conditions and then normalized to DMSO-treated conditions.

### Sensory neuron differentiation, maintenance and drug treatments

Human sensory neurons were differentiated from hiPSCs as previously described (Deng et al., 2022 preprint). In short, 1.5 million hiPSCs were plated in each well of a six-well plate until 90% confluence. Differentiation medium 1 [E6 medium (Thermo Fisher Scientific, A1516401) containing 0.2 µM CHIR-98014 (Sigma-Aldrich, SML1046-25MG) and 2 µM A83-01 (STEMCELL Technologies, 72022)] was used to feed the cells for 3 days. Cells were then re-plated and aggregated using AgreWell 800 plates (Corning) at 5.5 million cells/well and spheres were maintained in differentiation medium 2 [E6 medium containing 0.5 µM CHIR-98014, 2 µM A83-01 and 25 nM PD173074 (STEMCELL Technologies, 72164)] for 11 days. On day 14 of differentiation, spheres were dissociated using the Gentle MACS Dissociator and the MACS EB Dissociation Kit from Miltenyi Biotec. Cells were re-plated on 384-well dishes, pre-coated with Geltrex at 5000 cells/well and matured in differentiation medium 3 [DMEM/F12 with N2 (Thermo Fisher Scientific, 17502048), B27 (Thermo Fisher Scientific, 17504044), BDNF (Thermo Fisher Scientific, PHC7074), GDNF (Sigma-Aldrich, G1401) and NGF (Thermo Fisher Scientific, 13257-019)] for a further 14 days. All experiments were performed at day 28 of differentiation (day 0 representing the hiPSC stage). All putative rescue compounds (NAD, NMN, NAM and DSRM-3716) were added 2 h prior to BTZ treatment. Imaging of the neurites and neurite tracking was performed using the Incucyte live-cell imaging system.

### Statistics

All data are expressed as mean±s.e.m. Statistical analysis was performed using GraphPad Prism. A Brown–Forsythe test was first performed to determine whether groups had equal variance. All analyses showed equal variance. A two-tailed unpaired Student's *t*-test, one-way ANOVA or two-way ANOVA was performed for experiments with one group, two groups with one variable or two groups with multiple variables, respectively. The ANOVA was followed by a Dunnett's or Sidak's multiple-comparisons post hoc test. In all experiments, *P*<0.05 was considered significant.

## Supplementary Material

10.1242/dmm.049358_sup1 Supplementary information
