# Correction: Bortezomib-induced neurotoxicity in human neurons is the consequence of nicotinamide adenine dinucleotide depletion

**DOI:** 10.1242/dmm.052989

**Published:** 2026-05-28

**Authors:** Andrew R. Snavely, Keunjung Heo, Veselina Petrova, Tammy Szu-Yu Ho, Xuan Huang, Crystal Hermawan, Ruth Kagan, Tao Deng, Ilyas Singeç, Long Chen, Lee B. Barret, Clifford J. Woolf

There was an error in *Dis. Model. Mech.* (2022) **15**, dmm049358 (doi:10.1242/dmm.049358).

The name of the author Keunjung Heo was spelled incorrectly (Keungjung Heo). The correct name appears above, and the author contributions have been updated.

The authors apologise for this error and any inconvenience it may have caused.

