# Supplementary material for: Bortezomib-induced neurotoxicity in human neurons is the consequence of nicotinamide adenine dinucleotide depletion
Source: Dis Model Mech. 2022 Dec 8;15(12):dmm049358. doi: 10.1242/dmm.049358 (PMC9789399; doi:10.1242/dmm.049358)
Supplement: Supplementary information [file dmm-15-049358-s1.pdf]

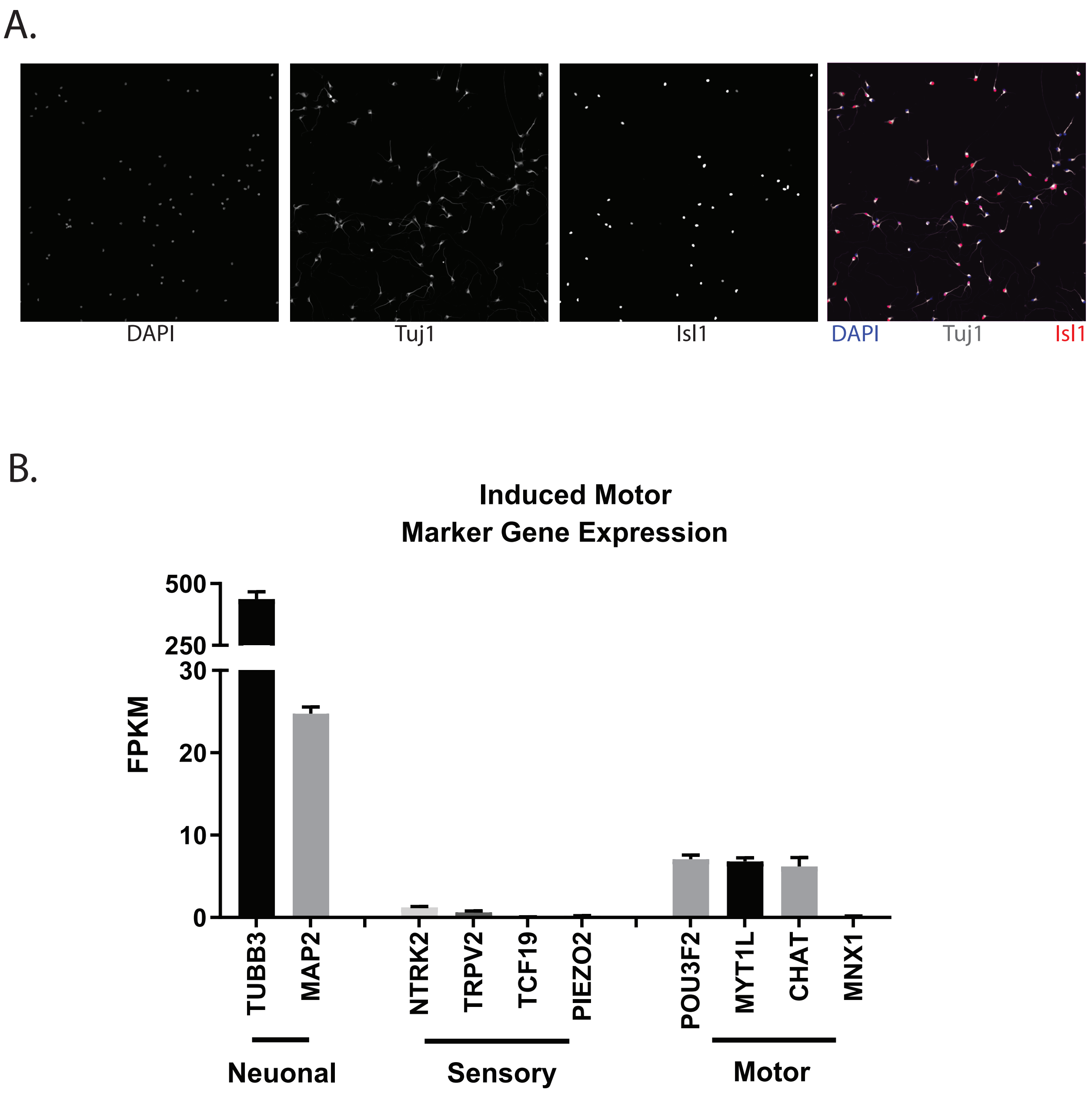

Fig. S1. Markers expressed by hiPSC-derived motor neurons. (A) Representative immunostaining of hiMN for the lineage marker Isl1. **(B)** FPKM values from RNA-sequencing of hiMN for a set of neuronal, sensory neuron, and motor neuron markers (n=3).

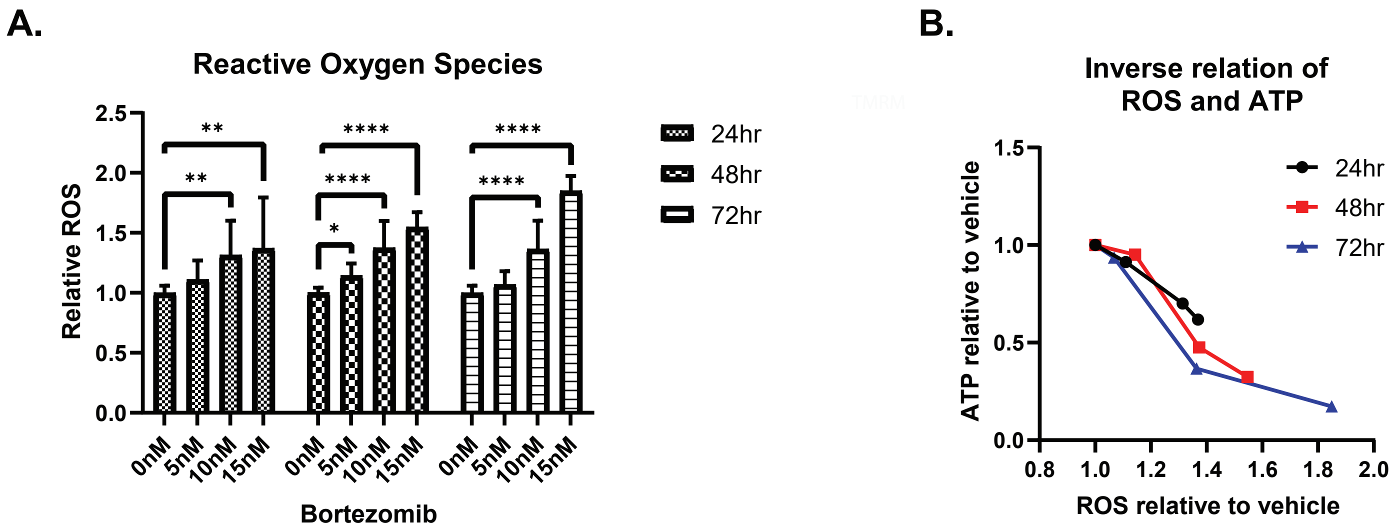

Fig. S2. Bortezomib treatment increases reactive oxygen species in hiMN. (A) hiMNs were treated with varying doses of BTZ and subjected to luminescence-based assays for ROS at 24, 46 or 72 hours. Data were tested with a 1-way ANOVA; post hoc Dunnet's multiple-comparisons test. *P < 0.05, **P < 0.005 , ***P < 0.0005 and ****P < 0.00005; (n > 14)**. (B)** Comparison of changes in ATP and reactive oxygen species over time in hiMNs treated with 15nM BTZ.

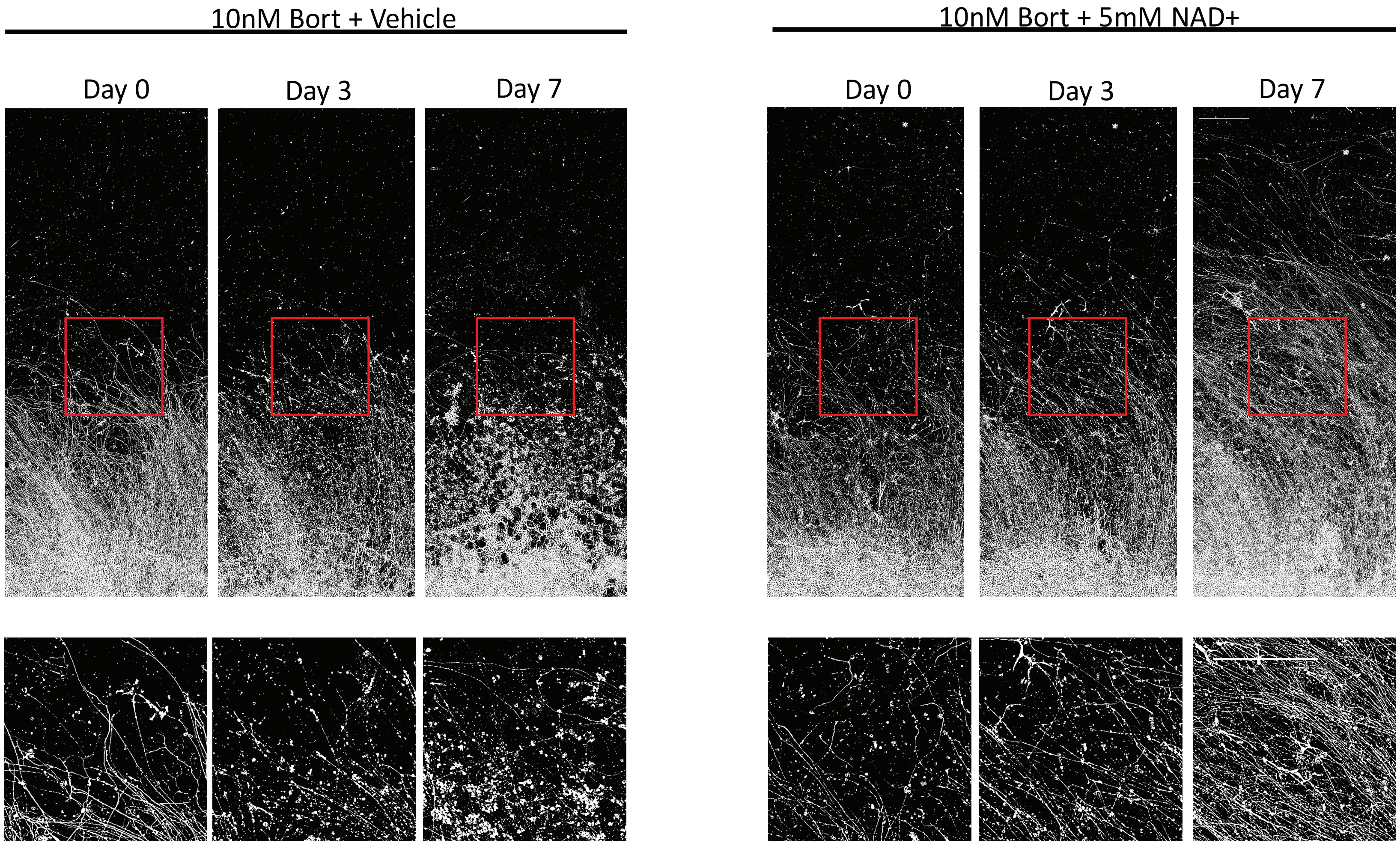

Fig. S3. NAD+ protects from bortezomib-induced neurite loss. Enlarged representative fields from representative images in Figure 4C. Scale bar represents 200 μM.

Disease Models & Mechanisms • Supplementary information

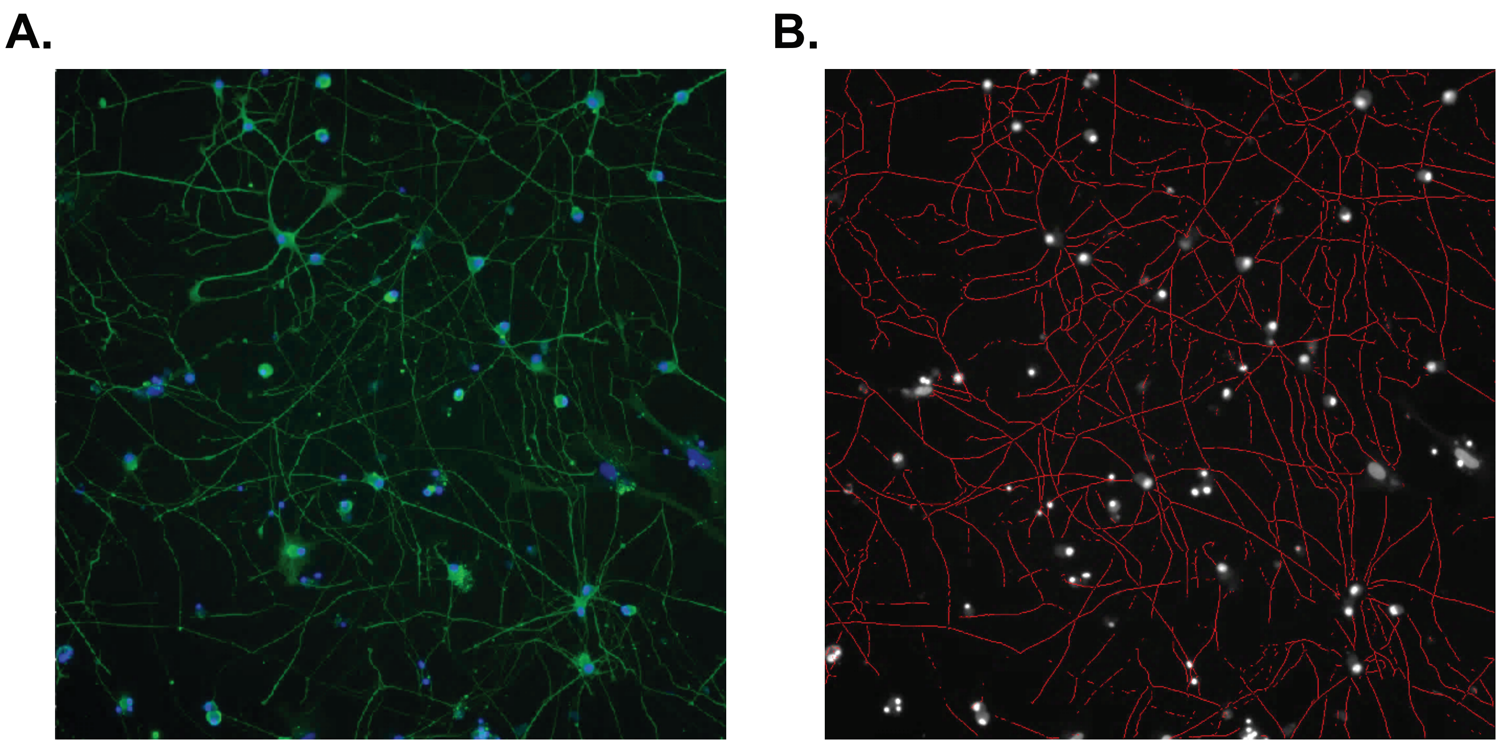

Fig. S4. Automated neurite tracing with ThermoFisher XTi Arrayscan. (A) Representative image of iMN grown in dissociated culture fixed and stained for β-tubulin (green) and DAPI (blue). **(B)** Neurite tracing (red) and nuclei (white) of iMN shown in (A).

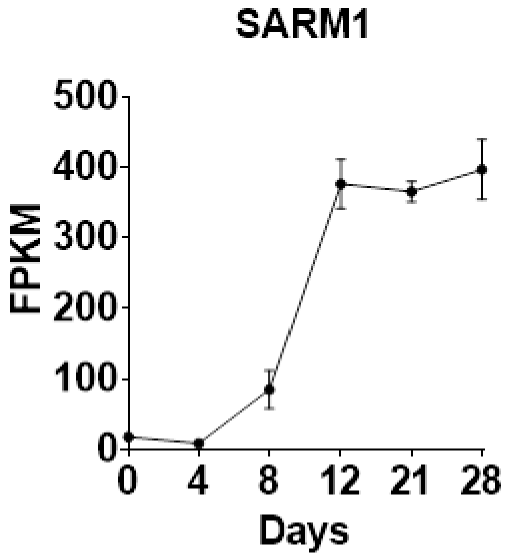

Fig. S5. SARM1 expression in iSNs. RNA-seq FPKM measurements of SARM1 expression in iSNs over time from day 0 to day 28 of differentiation. Error bars represent SEM, N=3
